# Ribonomics Approaches to Identify RBPome in Plants and Other Eukaryotes: Current Progress and Future Prospects

**DOI:** 10.3390/ijms23115923

**Published:** 2022-05-25

**Authors:** Muhammad Haroon, Rabail Afzal, Muhammad Mubashar Zafar, Hongwei Zhang, Lin Li

**Affiliations:** 1 National Key Laboratory of Crop Genetic Improvement, Huazhong Agricultural University, No. 1, Shizishan Street, Hongshan District, Wuhan 430070, China; muhammadharoon786@webmail.hzau.edu.cn (M.H.); rabail.afzal.10@gmail.com (R.A.); 2 State Key Laboratory of Cotton Biology, Key Laboratory of Biological and Genetic Breeding of Cotton, The Ministry of Agriculture, Institute of Cotton Research, Chinese Academy of Agricultural Science, Anyang 455000, China; m.mubasharzafar@gmail.com; 3 Institute of Crop Science, Chinese Academy of Agricultural Sciences, Beijing 100081, China

**Keywords:** RNA-binding proteins, ribonomics, RNA–protein interactions, transcriptome-wide RBPs

## Abstract

RNA-binding proteins (RBPs) form complex interactions with RNA to regulate the cell’s activities including cell development and disease resistance. RNA-binding proteome (RBPome) aims to profile and characterize the RNAs and proteins that interact with each other to carry out biological functions. Generally, RNA-centric and protein-centric ribonomic approaches have been successfully developed to profile RBPome in different organisms including plants and animals. Further, more and more novel methods that were firstly devised and applied in mammalians have shown great potential to unravel RBPome in plants such as RNA-interactome capture (RIC) and orthogonal organic phase separation (OOPS). Despise the development of various robust and state-of-the-art ribonomics techniques, genome-wide RBP identifications and characterizations in plants are relatively fewer than those in other eukaryotes, indicating that ribonomics techniques have great opportunities in unraveling and characterizing the RNA–protein interactions in plant species. Here, we review all the available approaches for analyzing RBPs in living organisms. Additionally, we summarize the transcriptome-wide approaches to characterize both the coding and non-coding RBPs in plants and the promising use of RBPome for booming agriculture.

## 1. Introduction

Owing to their sessile nature and fixed growing places, plants have evolved an adaptive mechanism to cope with changing environments [1]. This mechanism involves the interaction of RNA-binding proteins (RBPs) with RNAs to regulate the plant’s response to biotic and abiotic stresses [2]. Due to their widespread function, RBPs are considered the checkpoint to modulate the fate of cells, and they control cell activities including transcription, the generation of coding and non-coding RNAs, translation, RNA decaying, RNA turnover, and other regulatory mechanisms involved in the plant developments and responses to various kinds of stresses.

RBPs exert their function by forming a complex interaction with RNAs via single or multiple RNA-binding domains (RBDs) [3], such as RNA recognition motif (RRM), RNA-binding motifs (RBM) [4], hnRNP K homology domain (KH) [5], DEAD-box helicase domain [6], glycine-rich region, arginine-rich region, RD-repeats, and SR-repeats [7]. The RRM and the double-stranded RBM are the two most abundant RBDs that determine the recognition of RNA binding with RBPs. RRM is abundantly present in RBPs and is comprised of 75–85 amino acids [8]. Conclusively, the recognition of targeted RNA sequences by RBPs is achieved by these specific RBDs which are also considered vital regulators to determine the functioning of RBPs [9].

The available methods for studying RNA–protein interactions are mainly categorized into RNA-centric, protein-centric, in silico, and transcriptome-wide approaches [10]. In this review, we highlight the conventional and up-to-date technologies for studying both coding and non-coding RBPs in living organisms. Additionally, we also discuss the current status and prospects of ribonomics approaches which would be helpful for RNA biologists to understand the role of RBPs in plants.

## 2. Experimental Approaches to Dissect the RBPome

The selection of the identification methods for RBPs relies on the type of RNA molecule [11]. The protein-centric and RNA-centric approaches characterize the RBPs bound with the RNA of interest [12], and in silico methods employ various computational methods to predict RBPs [11]. In the past, the RBPome was not a popular research topic due to the instability and sensitivity of mRNA, and the polyadenylation dependency of RBPs. Recently, transcriptome-wide methods such as Protein-X-linked RNA Extraction (XRNAX), Click Chemistry-assisted RNA-Interactome Capture (CARIC), and Phenol Toluol Extraction (PTex), which do not require poly-A tail, have been successfully developed and used to study RBPome in living organisms. These transcriptome-wide approaches have been applied in several organisms including *Caenorhabditis elegans* and humans to identify both coding and non-coding RBPs [13].

### 2.1. Protein-Centric

**Figure 1:**
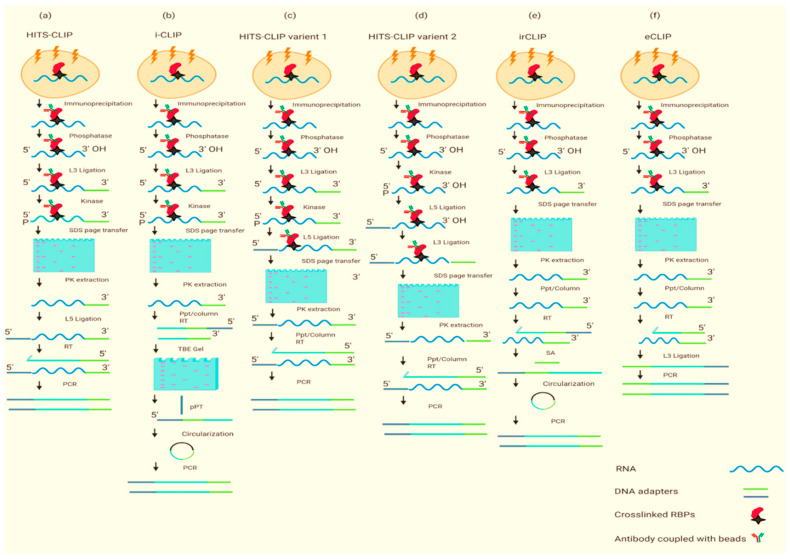
The above chart was modified from [11] in which (**a**–**f**) shows the graphical representation of CLIP-seq methods. Although all CLIP methods are not included, a detailed explanation is given in Table 1. XL, UV cross-linking; IP, immunopurification; phosphatase, removal of 3′ phosphate; kinase, the addition of 5′ phosphate; RT, reverse transcription; L3, 3′ adaptor ligation to RNA or DNA; L5, 5′ adaptor ligation; PK extraction, proteinase K extraction from nitrocellulose membrane; Ppt/column, alcohol precipitation or column cleanup of nucleic acid; TBE, Tris–borate–EDTA; SA, streptavidin.

Protein-centric methods aim to identify RNAs that bind to proteins of interest [14]. Interacting RNAs that bind to the protein of interest are firstly reverse transcribed into cDNA, PCR amplified, and sequenced [14], and further bioinformatics tools are used to identify the RBP binding sites [15]. A breakthrough in protein-centric methods occurred with the development of cross-linking immunoprecipitation (CLIP) [16], and some CLIP variants (Figure 1) have been employed in *Arabidopsis* to identify the mRNA targets [14,17]. Recently, a new protein-centric method, Hyper Targets of RNA-Binding Proteins Identified by Editing (HyperTRIBE) was developed to identify RBPs targets in plants [17]. Compared to RIP-seq and CLIP, HyperTRIBE is more efficient for the small number of samples [17].

**Figure 2:**
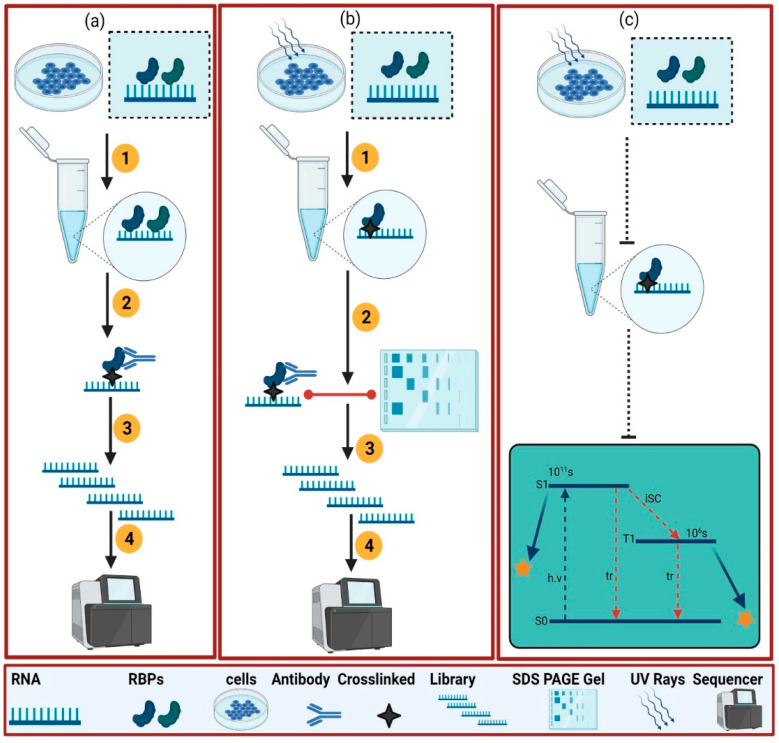
This illustrates the protein-centric method applied in eukaryotes. For the protein-centric method, two kinds of variants are generally used: native purification (**a**) and the denaturing method (b) [14]. (**a**) is showing the native purification method which is called RNA immunoprecipitation (RIP). It consists of several steps to experiment with call lysate containing targeted and other RBPs (1), capturing of targeted RBPs with a specific antibody (2), RNA library preparation (3), and sequencing (4). (**b**) works based on UV cross-linking and immunoprecipitation which is called the crosslinking immunoprecipitation (CLIP) method. In the (**a**) method, a specific protein of interest is immunoprecipitated from a cell by a specific protein antibody in the presence of native conditions; UV cross-linking of cells as shown by the star for binding the RBPs and RNA (1), capturing of specific RBPs using an antibody and SDS-page gel analysis confirmation (2), preparation of the associated RNA library (3), and sequencing (4). (**c**) shows a simplified Jablonski diagram [18] and explains the excitation of RNA. During the UV cross-linking mechanism, RNA goes from the ground state (S0) to the excited state (S1). During the procedure, the inter-state conversion (isc) to a triplet (T1) state happens. S1 and T1 are the excited states which fall back to the ground state in 10 (ps) and 1 (µs), respectively. They fall back to the ground state either in the form of thermal relaxation (tr) or the crosslinking formation of RNA with the adjacent amino acid (yellow star).

To overcome the drawbacks associated with the “native purification methods (Figure 2a)”, a “denaturing method” (Figure 2b) has been developed. In this method, RNAs are cross-linked with the RBPs by different UV cross-linking rays [14]. In this treatment, both RNA and DNA absorb the 254 nm UV light efficiently and are excited to the higher energetic states S1 and T1, respectively (Figure 2c) [18]. Upon UV cross-linking, a specific type of physical bond is formed between RNAs and proteins. UV cross-linking has several advantages and disadvantages. Its advantages include the interactions of frozen RBPs [18], the zero-distance interaction between RNA and RBPs [19], and the formation of a stable covalent bond which is retained while washing under stringent conditions [18]. Disadvantages are the low binding efficiency (only 5%) [18], interactions of a single transcript with multiple proteins [20], low UV cross-linking efficiency in the presence of tissues, turbid liquid cultures [20,21], and the formation of unnecessary interactions such as RNA–RNA, RNA–DNA, and protein–protein interactions [18]. However, the UV cross-linking inefficiency can be circumvented by optimizing the UV cross-linking rays [22]. Usually, a short wavelength UV light is utilized to ensure the efficient cross-linking of RNA with RBPs.

### 2.2. RNA-Centric Approaches

RNA-centric approaches are used to identify proteins that bind with the RNA of interest [11]. Generally, most of the currently available methods use tagged RNA as bait to capture the cross-linked RBPs, and the identified RBPs are characterized using mass spectrometry [23]. RNA-centric approaches are categorized into two main variants: in vitro and in vivo variants (Table 1).

**Table 1:** Protein-centric approaches to find the targets of RBPs.

Method	Advantage	Disadvantage	Ref
PAR-CLIP, HITS-CLIP	Efficient, RBPs can be identified at the 3′ end of RNA, highly specific	UV cross-linking does not bind the RNA proteins well which can lead to a high number of false positives, crosslinked nucleotides are bypassed by reverse transcriptase, it is a laborious and time-consuming protocol	[24,25]
iCLIP	An efficient method that does not need the reverse transcription to bypass the bound nucleotides, high resolution	UV cross-linking does not bind the RNA proteins well, interactions near the 3′ end of an RNA may be unidentifiable because reverse transcriptase stops at the cross-linked nucleotide, unable to identify RNA–protein interaction near 3′ RNA end as reverse transcription stops at the crosslinked nucleotide	[26]
HITS-CLIP variants 1 and 2	A short method, more reliable as both ligations are carried out by beads, genome-wide	Low efficiency of UV cross-linking, crosslinked nucleotides are bypassed by reverse transcriptase	[27]
eCLIP,	It does not need a circularization step which makes it unreliable, decreases requisite amplification by ~1000-fold	Low efficiency of UV cross-linking, time-consuming protocol, single-stranded DNA adapter is obligatory for the ligation to single-stranded cDNA.	[27]
sCLIP	Simplified, robust, permits a radiolabel-free visualization of immunoprecipitated RNA, improves the complexity of the sequencing library	Low efficiency of UV cross-linking, time consuming	[28]
irCLIP	Efficient, use of fluorescent adapter at each step to visualize crosslinked RNA	Low efficiency of UV cross-linking, time-consuming protocol, unable to identify RNA–protein interaction near 3′ RNA end as reverse transcription stops at the crosslinked nucleotide	[29]
GoldCLIP	Less time-consuming protocol, gel purification is not necessary	Low efficiency of UV cross-linking, expression of the fusion protein is necessary, unable to identify RNA–protein interaction near 3′ RNA end as reverse transcription stops at the crosslinked nucleotide	[30]
fCLIP	Use of formaldehyde instead of UV cross-linking, higher efficiency for developing the interaction between double-stranded RNA and proteins	Time-consuming protocol, formaldehyde crosslinking is not as good as UV cross-linking	[31]
BrdU-CLIP	Efficient for removing the “empty adapter” reads during reverse transcription because these reads can clutter HITS data	Low efficiency of UV cross-linking, time-consuming protocol, unable to identify RNA–protein interaction near 3′ RNA end as reverse transcription stops at the crosslinked nucleotide	[32]
TRIBE	The protein of interest is not purified for this protocol, no use of UV cross-linking, RBPs can develop interaction at any site of RNA	Not very effective in RBPome, it was not used for many studies	[33]
CRAC	Efficient due to the two-step affinity purification of tagged proteins in yeast, completely removes any interaction RBPs, RNAs that are not crosslinked to the protein of interest	Only works under denaturing conditions, very challenging, time-consuming, long protocol	[34]
RNA tagging	The protein of interest is not purified for this protocol, no use of UV cross-linking, RBPs can develop interaction at any site of RNA, easy protocol	Only studied in *Saccharomyces cerevisiae*, does not work well for the RBPs which are away from the 3′ RNA end	[35]
HyperTRIBE	Simple as a comparison to other protein-centric methods to identify the targets of the RBPs	A lot of validation steps are involved	[17]

#### 2.2.1. In Vitro Methods

The in vitro approach involves the biosynthesis of RNA bait, the binding of the tagged-RNA to resin, the formation of a complex of ribonucleoproteins (RNPs), and the washing, purification, and elution of the RBPs that are bound with the tagged RNA [36,37]. The in vitro method is sensitive to a few challenges: in vitro transcribed RNAs have different structures and modifications from normal RNAs, the association of RBPs with RNA which might not occur due to the lack of posttranslational modifications, and the formation of an anomalous complex such as heterogeneous nuclear ribonucleoproteins (hnRNPs) [38].

**Figure 3:**
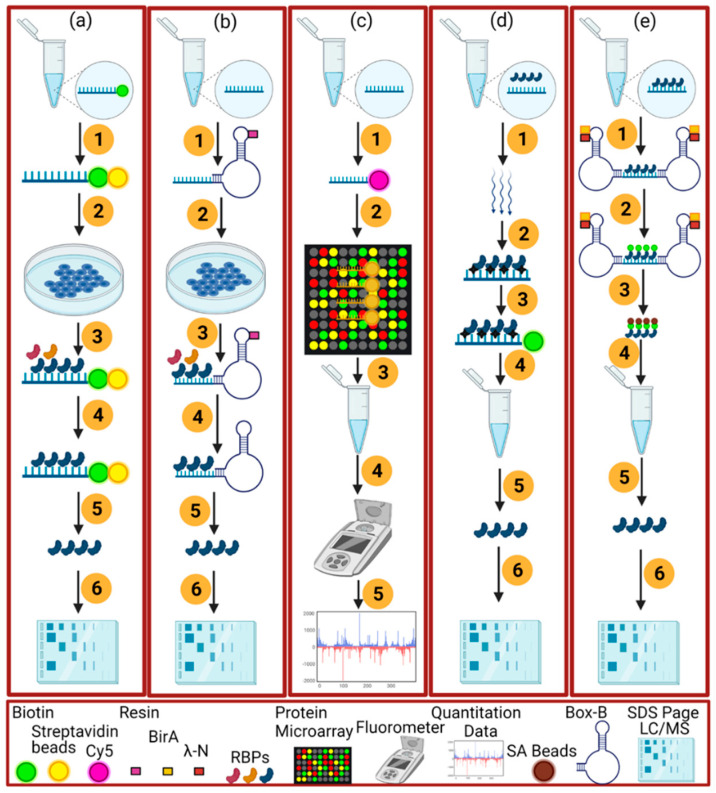
The graphical representations of the in vivo (**a**–**c**) and in vitro (**e**,**d**) RNA-centric methods. (**a**) the schematic of the end-biotinylated-RNA pulldown method which consists of these steps; biotin tagged RNA is transcribed in vitro and incubated with streptavidin beads (1), the addition of a cellular extract (2), the connection of RBPs with the associated RNA (3), non-targeted RBPs are washed (4), boiling of beads and RNase treatment to elute the RBPs (5), running of SDS-Page gel and mass spectrometry (6). (**b**) an aptamer-tagged RNA method to identify the RBPs in in vivo by not using the UV cross-linking mechanism as in (**a**). It consists of these steps, the linking of in vitro transcribed RNA with an RNA tag (blue) and resin (1), binding of RBPs with the RNA upon the addition of a cellular extract (2, 3), washing of specific and non-targeted RBPs (4), elution of the RBPs by using imidazole for Cys4 or biotin for the S1 aptamer method (5), LC/MS (6). (**c**) a protein microarray method followed by: in vitro RNA is transcribed by using the Cy5 (1), complex formation of RNA with the spotted 9400 proteins (2), washing of proteins (3), use of fluorescence meter to quantitate RNA bound with the microarray proteins (4, 5). However, (**d**) an in vitro UV cross-linking-based method as in plants (RIC, eRIC, OOPS) or other eukaryotes (RAP, PAIR, MS2-BioTRAP, TRIP). It consists of the UV cross-linking of RBPs inside the live cell (1, 2) the capturing of RBPs with the biotin (3), purification and isolation of RBPs (4, 5), SDS-Page gel and LC/MS (6). In the last (**e**), the non-UV cross-linking method RaPID is carried out by the following procedure: flanking of RNA with the BoxB RNA stem-loops (1), biotinylation of proteins by using the RaPID (LN-HA-BirA*) fusion protein linking with BoxB sites (2), capturing of biotinylated proteins with the SA beads (3), washing and elution of RBPs (4, 5) and SDS-Page Gel and LC/MS (6).

The major drawback of the in vitro RNA-tagged method is the alteration of the secondary structure of RNA due to its interaction with the labeling dyes [39]. For RNA labeling, the used chemicals include biotin, fluorescent dyes, digoxigenin, and many unlisted compounds [11,39]. The common RNA labeling is biotinylation (Figure 3a), in which the 5′ or 3′ end of RNAs are biotinylated based on the “RNA pull-down method” [40]. Further, upon the addition of streptavidin beads, the biotinylated RNA bound to proteins in the cellular extract forms an immobilized complex. Subsequently, RNA-bound beads are washed and boiled to remove the non-specific RNA–protein interactions [41,42].

To circumvent the drawbacks of different dyes, numerous natural and artificial aptamers are used to increase the affinity of RNA with the proteins [11,41]. Streptavidin-binding aptamer (S1 aptamer) tags have emerged as useful tools to identify the specific RNPs [41]. These aptamers have a high affinity toward immobilized streptavidin beads and are highly stable even in the presence of high salt conditions (400 mM NaCl) [41]. After S1 aptamer tags bind to streptavidin beads, biotin is added to bind with streptavidin beads, and the binding elute RNA is tagged by the S1 aptamer from the cellular extract [43,44]. Doudna and colleagues used cys4 endoribonuclease (Figure 3b) [42] to isolate the RNPs accurately. The incorporation of cys4 endoribonuclease makes a sticky interaction between RNPs and with the tagged cys4 hairpin loop, facilitating the cleavage of RNPs. Further addition of imidazole allows the cys4-endoribonuclease to break the cys4 hairpin loop and liberate the RNA–protein complex with a high specificity [11,45].

Protein microarray is also an alternative in vitro approach [11]. In this approach (Figure 3c), cy5 dye is used to label the RNA of interest and is followed by RNA hybridization with the recombinant proteins. The major drawback of this approach is the changes in folding and the posttranslational modifications of recombinant proteins, and the artificial concentration of proteins may distort the interaction [11].

Generally, in vitro methods work specifically and efficiently for the individually known RNAs. However, it is difficult to know the insertion place of natural and artificial aptamer tags without any structural information about the RNA of interest, and the insertion of tags changes the RNA structure. Moreover, these aptamers are not resistant to endonucleases which could reduce the lifetime and recovery rate of RBPs [43,46]. The addition of different dyes and aptamers tags distorts the chemical properties of RNA–protein interactions. To alleviate the challenges caused by dyes and aptamers, F. Ataide and his colleague developed the “Antisense RNA capture” method to isolate and identify the RNP complexes. The streptavidin–biotin interaction is employed to immobilize the affinity-tagged antisense oligonucleotides, and later the RNA of interest is hybridized with the antisense oligonucleotide; thus, the associated protein complex is isolated. Owing to the high stability and strong binding and hybridization with the RNA, this method is used to study various complexes of RNPs, including snRNA and telomerase RNA–protein complexes [39]. In this method, there is no need to label the bait RNA or the RNA of interest. However, it is a very challenging task to design the antisense oligonucleotide to detect the RNA of interest [44].

#### 2.2.2. In Vivo Method

The in vivo method isolates and identifies the RNA–protein complexes inside the cell and retains the integrity of RNA–protein complexes by developing a strong covalent bond between them. The in vivo method has two variants depending on whether UV cross-linking is needed [11,45,46]. The UV-crosslinking method purifies the RBPs inside the cell under denatured conditions (Figure 3d), and can remove non-specific or non-covalent bonded proteins [47]. UV cross-linking can only bind RNA and proteins at zero distance [48]. Short wavelength UV radiations (254 nm) develop a strong covalent bond between the RNA and the protein [43]. Two research groups employed the UV-based RIC approach to identify the mRNA-bound proteome from human embryonic kidney cells and human HeLa cells. In plants, the method was used for the identification of RBPs in *Arabidopsis* [49]. However, low RNA abundance is a big challenge in the identification of RNA–protein interactions [1,14,50]. Moreover, RIC neglects non-coding RNAs [51].

The other variant of the in vivo method is formaldehyde cross-linking, which links together the macromolecules within 2 Å. Formaldehyde cross-linking can form protein–protein, RNA–protein, and DNA–protein interactions, and works efficiently on cells, tissues, and even whole organisms [47]. Formaldehyde crosslinking has different biases: nucleophilic lysine residues are strongly prone to form cross-linkages [52]; promotes nonspecific interaction such as DNA–proteins and protein–protein [53]; low cross-linking efficiency requires a significant number of cells (~10^8^−10^9^) [54]; many proteins can bind with the same RNA transcript [14].

There are RNA-centric variants that do not need UV and chemical-based cross-linking. “Promiscuous” biotin ligase (BioID) is one of these RNA-centric variants [50]. In this method, biotin is converted to reactive biotin-5-AMP, an intermediate that covalently labels the targeted protein and any nearby proximal proteins [55]. Biotin-5-AMP possesses a quenching behavior and becomes reactive within a distance of 20 nm to its point of release, and labels all of the nearby proteins [36]. Before applying this method, the RNA of interest is tagged with BoxB aptamers for recruiting the RaPID (LN-HA-BirA*) fusion protein (Figure 3e). The linked BoxB aptamer with the fusion protein not only biotinylates the targeted protein, but also the nearby proteins proximal to the RNA. Later, streptavidin beads are used to isolate the biotinylated RNPs for further proteomic analysis [11]. Despite cons such as simplicity and timesaving, BioID has a few pros including the BoxB site being proximal to RNA of interest; the artificial expression of bait RNA by plasmid transfection; and the formation of complex structures due to RNA folding. It needs to be careful about the positioning of the BoxB aptamer in the case of longer RNA species because it works efficiently for shorter (≤132 nt) RNA motifs [11].

Despite the availability of several RNA-centric approaches, only the RIC RNA-centric approach was employed in *Arabidopsis* [22,37,49,56,57,58,59,60].

## 3. In Silico Approaches

Both protein-centric and RNA-centric approaches are useful for the identification of RBP in humans, yeast, and plants [49]. However, many of these approaches are time-consuming, costly, and uncontrollable [39]. In silico approaches arise with the accumulation of a large amount of public protein data. In silico approaches use computational methods for the annotation and elucidation of the RNA–protein complexes [39].

Mainly, computational methods are categorized into two categories: template-based and machine learning methods. Template-based methods, initially, find sequence similarity between query and template (known to bind RNA) for assessing the RNA binding preference of the protein sequences, whereas the machine learning method creates predictive models that can find a pattern in the input feature space to score the probability of the RNA-binding preference. Various features and algorithms are used in the machine learning approach for deciphering the RNA–protein interactions [61,62]. Some commonly used approaches have been discussed in detail for the identification of RBPs. AIRBP is one advanced machine learning approach [39,63]. In AIRBP, “Stacking” is used to predict the RBPs, in which different features are extracted from physiochemical properties, disordered properties, and evolutionary information [64], and used to train the predictive model [63]. However, in silico approaches are devised based on the in vitro methods which determine the set of obtained positive sequences or the structural information of RNAs.

## 4. Transcriptome-Wide Identification of RBPs

**Figure 4:**
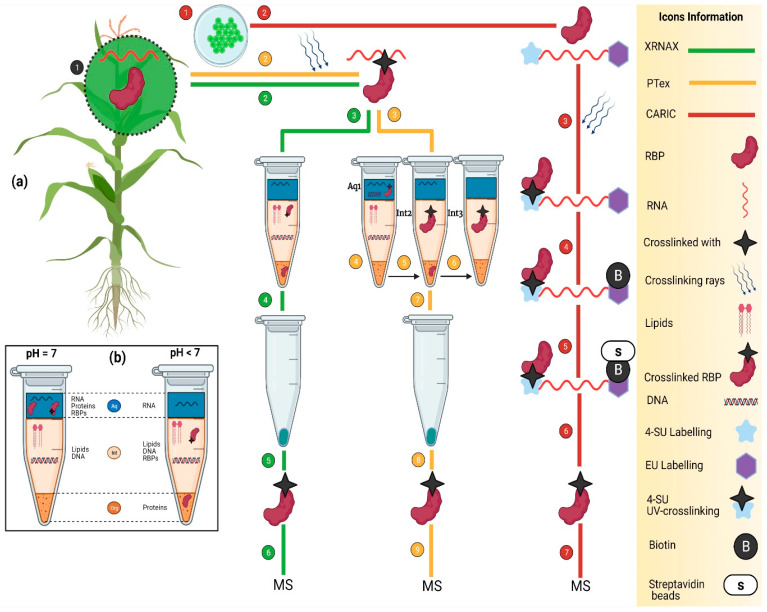
(**a**) the schematic representation of transcriptome-wide approaches to identify the RBPs. (Green line) is an XRNAX method that consists of a series of steps including plant growth (1), UV cross-linking (2), interphase aspiration, washing and DNase digestion (3), RBPs pellet collection (4), RBPs ready for MS (5), MS analysis (6). (Yellow Line) is the PTex method which works based on a differential pH as demonstrated in (**a**). It consists of a series of steps to isolate the RBPs including plant growth (1) and UV cross-linking (2). Three further differential enrichment steps are performed as follows: Phenol and Toluene (pH 7, PT 50:50); acidic phenol; and phenol, ethanol, and water (pH 4.8) numbered as (3–6), respectively. As a result of the differential enrichment scheme, RBPs are enriched as follows: aq1 > int2 > int3 in (4–6). In a final step (7), ethanol is added and centrifuged to precipitate the RBPs in pellet form. (Red line) shows the CARIC strategy in which plant cells (1) are grown in the presence of 4SU (sky blue star) and EU (purple star) (2), and several RNAs uptake them. As a result of UV cross-linking (365 nm), 4SU is activated and cross-links RNA with binders (black star) (3). After cell lysis, cells are labeled with biotin to tag the EU (4). Further RNase digestion and streptavidin bead-enrichment steps are performed to digest the RNA and release RBPs, respectively (4 and 5). RBPs are isolated (6) and ready for MS (7). (**b**) the description of RBP-enrichment at varying pH levels. At pH 7 and <5, RBPs are enriched in aqueous and interphase, respectively.

In plants, several studies have been conducted for the transcriptome-wide identification of coding RBPs by employing the RIC method [22,49,59,65,66], which identifies the RBPs linked to polyadenylated poly(A) RNAs. Several efficient RBPs identification methods have been developed which are available to isolate the genomewide RBPs in plants, including XRNAX [51], CARIC [13], and PTex (Figure 4) [67].

### 4.1. XRNAX

To overcome the drawback of RIC, Jackob and colleagues developed the XRNAX method for the identification of transcriptome-wide RNA binding proteins [51]. XRNAX features UV cross-linking apparatus that cross-links RNA and proteins. XRNAX can isolate the coding and non-coding RBPs, regardless of whether RNAs are polyadenylated or not [51]. However, in XRNAX, it is compulsory to optimize the UV cross-linking because only 5% of the proteins are cross-linked with the RNAs [1]. For the successful identification of RBPs, the minimum amounts of 8 × 10^7^ cells are required for RBP enrichment [68]. By employing the XRNAX method, more than 700 non-polyadenylated-linked RBPs and WKF RBDs were identified [51]. Besides RBP identification, all biotypes of cross-linked RNAs (both coding and non-coding) were identified using XRNAX [51].

### 4.2. PTex

Beckman and colleagues developed PTex (Figure 4b) for the identification of transcriptome-wide RBPs [67]. PTex relies on physiochemical properties and identifies all kinds of RBPs including proteins that interact with short RNAs (30 nt). PTex requires a fewer amount of cells (∼5 × 10^6^ cells) than the RIC approach [69]. Practically, PTex has been used for identification of the RNA-bound proteome of human HEK293 cells and the bacterium *Salmonella Typhimurium* [67].

### 4.3. CARIC

Similar to XRNAX and PTex, the CARIC method captures both poly(A) and non-poly(A)-dependent RBPs. It consists of a series of steps: the metabolic labeling of RNAs (mRNA and non-coding RNAs) with 4-thiouridine (4SU) and 5-ethynyl uridine (EU); in vivo RNA protein photo cross-linking; reaction with azide-biotin; use of biotin tags for the affinity enrichment; and isolation of RBPs by streptavidin beads. Due to its universal acceptability towards the eukaryotes, CARIC has been used in living organisms such as bacteria [13], animals, and plants. For example, CARIC identified 597 known RBPs in HeLa cells including 130 novel RBPs [13]. However, because CARIC was restricted to cross-linking with RNA that had an alkynyl uridine analogue, it identifies fewer RBPs than PTex, XRNAX, and OOPS [70].

### 4.4. OOPS

OOPS retrieves the free protein, protein-bound, free RNA, and cross-linked protein RNA in an unbiased manner, and enables the study of each component of the RNA–protein complex separately [71]. It does not need molecular tagging or the capturing of polyadenylated RNA. OOPS starts with the UV cross-linking of the RNAs and proteins, and RBPs are separated in phase separation according to their gradient. The required components for analysis can be retrieved using protease digestion to digest protein in protein-bound RNA or using the digestion of RNAs to digest RNA in RBPs [72,73]. The organic phase separation requires a lower amount of ∼3 × 10^6^ cells for RBP-enrichment in OOPS [73]. OOPS can capture unique RBPs that are not identified by any other methods [72,73], and is the first method to identify the transcriptome-wide RBPs in plants.

## 5. Identification of RBPs in Plants

Recent discoveries have revealed that RBPs have various functions and have shown great implications for crop improvement [7,74]. For example, the expression of both AtGRP2 and AtGRP7 proteins conferred a higher grain yield than the control lines under salt and cold stresses [75]. GRP8 is responsible for the phosphate uptake and biomass accumulation and can be edited to increase the phosphate uptake and utilization in plants [76], and MhGR-RBP1 showed high transcript levels in response to several abiotic stresses [77]. Furthermore, it was found that some RNA chaperones can make a plant resistant to external cues. For example, expressing a cold shock protein in maize showed a 6% increase in the yield in field trials under drought stress conditions [78]. The expression of AtRGGA conferred resistance against osmotic stress in response to ABA salt stress in *Arabidopsis* [79], while the overexpression of RBP MhYTP1 increased the drought resistance in apples [80]. The above-cited literature indicates that RBPs can be exploited in the improvement of plant traits.

**Figure 5:**
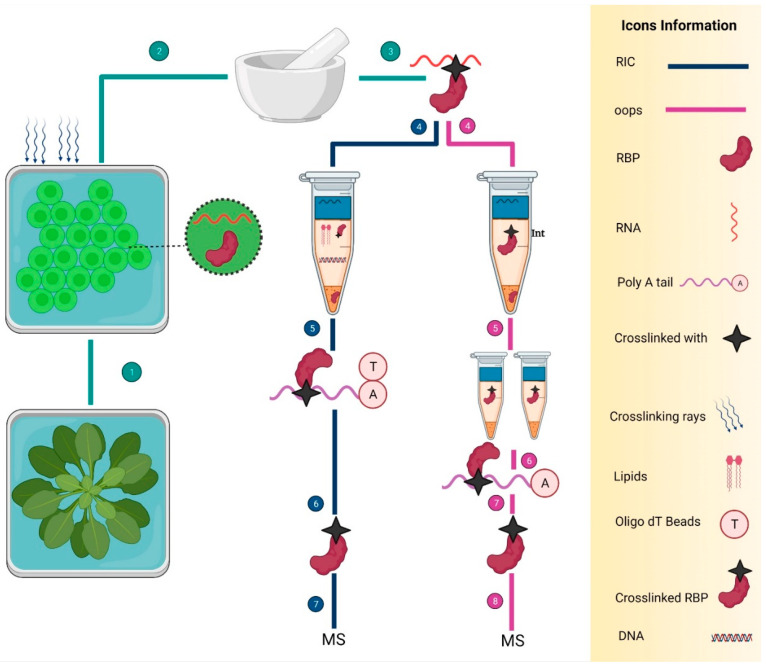
Two ribonomics methods to identify the RBPs in *Arabidopsis*. Navy blue and pink colored lines show the RIC and OOPS, respectively. On the right side, there is detailed information about the icons. (1), (2), and (3) are the common steps that show the cutting of *Arabidopsis* leaves into the tiny round-shaped cuttings, UV cross-linking, and grinding small cut leaves in liquid nitrogen, respectively. Next, all the steps are specific in both methods. RIC consists of a total of seven key steps: the extraction of UV cross-linked RBPs (4), employment of oligo-d(T) beads to capture the mRNA (5), RNase treatment to eliminate the RNA (6), and mass spectrometry for the sequencing of RBPs (7). However, the OOPS also consists of seven steps: isolation of RBPs from the interphase by using acidic guanidinium-thiocyanate-phenol (Trizol) and (Trizol: chloroform = 5:1 (*v/v*)), respectively (4), two-time purification of RBPs by the AGPC phase separation cycles and cryogenic treatment (5) RNase treatment to eliminate the bound RNAs (6), RBPs separation (7), and mass spectrometry (8).

Among the ribonomic approaches available to identify RBPs [13,51] in other living organisms including humans, animals, and microbes, a few approaches have been modified for application in plants. For example, RIC, RIP-seq, and CLIP-seq [59] have been employed in *Arabidopsis* (Table 2), leading to the identification of hundreds of RBPs and their interacting RNAs. Recently, the application of RIC in 2020 [22] and OOPS in 2020 (Figure 5) provided the landscape of the RBPome in plants by identifying all coding and non-coding covalently linked RBPs [81]. Further utilization of ribonomics methods devised for bacteria and animals would accelerate the identification and characterization of RBPs in kingdom plantae and would facilitate the study of the role of RBPs in plants.

## 6. Conclusions and Future Prospects

As a result of the above-stated conventional and modern ribonomics approaches such as RIC, CLIP, PtRIC, XRNAX, PTex, and CARIC, a wide range of coding and non-coding RBPs have been identified [13,55,65,73]. The above-stated transcriptome-wide approaches have been applied in mammalian cells and identified thousands of RBPs [13,65,73]. These transcriptome-wide approaches can also be employed in plants by optimizing the established protocols.

Why is the identification of RBPs is important? In short, RNA and protein interactions are everywhere in living organisms and play a role in changing the fate of cells by regulating gene expression. It is compulsory to understand the regulatory roles of transcriptome-wide RBPs. Methods such as XRNAX, RIC, CARIC, and OOPS should be prioritized in plants. RNA biologists could also complement the interactome capture technologies such as RIC, eRIC, and ptRIC [59] with the other protein-centric approaches such as iCLIP, HITS-CLIP, and others in the future [58].

## Figures and Tables

**Table 2:** All earlier ribonomics approaches devised in mammals and applied in plants.

Method	Purpose	Plant Specie	Identified RBPs/RNAs	Ref
RIC	Discovery of the RNA-binding proteome of plant leaves with an improved RNA interactome capture method	*Arabidopsis*	717	[22]
RIC	Determination of the mRNA-binding proteome of *Arabidopsis* etiolated seedlings	*Arabidopsis*	700	[82]
RIC	It was used in cells from different ecotypes grown in cultures and leaves to find the RBPs	*Arabidopsis*	1145	[65]
RIC	To capture the mRNA interactome from plant protoplasts	*Arabidopsis*	325	[56]
PtRIC	To check the change in RBPs in response to environmental cues	*Arabidopsis*	717	[22]
RIC	To check the effect of severe drought stress on the RNA-binding proteome	*Arabidopsis*	1408	[60]
RIP-seq	To identify the RNAs bound with the protein of interest	*Arabidopsis*	4000	[65,83]
iCLIP-seq	To unravel genome-wide RNA–protein interactions in vivo and the landscape of AtGRP7	*Arabidopsis*		[14,17]
HITS-CLIP	To study the function of RBP (HLP1) in flowering by targeting alternative polyadenylation	*Arabidopsis*		[84]
OOPS	To find all RBPs in plant extracts (both coding and non-coding)	*Arabidopsis*	468	[81]

## Data Availability

Not applicable.
